# Clinical profile of cerebral venous thrombosis and the role of imaging in its diagnosis in patients with presumed idiopathic intracranial hypertension

**DOI:** 10.4103/0301-4738.77025

**Published:** 2011

**Authors:** Suneetha Nithyanandam, Mary Joseph, Thomas Mathew

**Affiliations:** Department of Ophthalmology, St John’s Medical College, Sarjapur Road, Bangalore - 560 034, India; 1 Neurology, St John’s Medical College, Sarjapur Road, Bangalore - 560 034, India

Dear Editor,

We read with interest the article on cerebral venous thrombosis (CVT) and idiopathic intracranial hypertension (IIH) by Agarwal *et al*., and wish to make the following comments.[1]

About 40% of all cases of CVT present with a syndrome of isolated intracranial hypertension (IICH), while the remaining 60% present with encephalopathy and focal deficits; in the antibiotic era the fourth rather uncommon presentation is cavernous sinus thrombosis.[2,3] The syndrome of IICH due to CVT mimics IIH closely. To differentiate these two conditions (CVT from IIH) Friedman has modified Dandy’s criteria.[4] The modification includes the necessity for magnetic resonance imaging (MRI) of the brain to rule out CVT in typical patients of IIH and gadolinium-enhanced magnetic resonance venography (MRV) in atypical IIH patients, like children and young adult males.[4]

At our center which is a multispecialty tertiary care private medical college hospital we have routinely been using MRI with and without MRV since 2000 in all cases of IIH. We found 30/82 (36.5%) patients with IIH-like clinical picture to have CVT, in contrast to 11% reported in this study, by Agarwal *et al*. This lesser diagnosis of CVT in IIH patients may be due to non-use of MRI routinely. In fact MRI is the standard of care in all brain syndromes, with computed tomography (CT) being done only in the presence of severe cost constraints. In fact it is economical to consider MRI as the primary imaging modality, as equivocal findings on CT would warrant additional MRI and cost to the patient.

As the report by Agarwal *et al*., is from a tertiary care ophthalmic institute the patients would have primarily been referred from a neurology unit for ocular complaints. It would have been interesting to know the occurrence of visual loss and ocular manifestations in the cohort presented. Severe visual loss is often the primary presentation of both CVT and IIH.[5] At our institute we are in the process of evaluating the occurrence of visual loss and visual outcome in CVT patients. In our patients we have documented visual field loss of varying severity in 31/60 (52.5%) patients with CVT; while the occurrence of visual loss in IIH patients in the same time period was 12/52 (23.1%). The visual loss in CVT is more acute and less amenable to treatment than visual loss in IIH.[5] Nevertheless all patients with CVT require regular monitoring of visual function, both central acuity and visual field analysis to prevent/reduce irreversible visual loss.
